# Tripartite motif protein 6 promotes hepatocellular carcinoma progression via multiple pathways

**DOI:** 10.55730/1300-0144.5668

**Published:** 2023-02-28

**Authors:** Yifeng ZHANG, Lin YUAN, Shanshan CUI, Song WU

**Affiliations:** 1 Division of Digestive System, Department of Internal Medicine, The Second Affiliated Hospital, Shandong First Medical University, Tai’an, China; 2 Division of Digestive System, Department of Internal Medicine, The 960th Hospital of PLA, Tai’an, China; 3 Division of Digestive System, Department of Internal Medicine, The First People’s Hospital of Tai’an, Tai’an, China; 4 Division of General Surgery, Department of Surgery, The Second Affiliated Hospital, Shandong First Medical University, Tai’an, China

**Keywords:** Hepatocellular carcinoma, prognosis, proliferation, tripartite motif containing 6

## Abstract

**Background/aim:**

Hepatocellular carcinoma (HCC) ranks among the most prevalent malignancies worldwide and the third leading cause of cancer-related death. The TRIM (tripartite motif-containing) protein family members had been reported to be involved in carcinogenesis and tumor progression. Here we aimed to explore the expression profile of TRIM6 in HCC and investigate its clinical significance as well as underlying mechanisms.

**Materials and methods:**

We retrospectively enrolled 138 HCC patients that underwent surgical resection in our hospital and tested protein expression level of TRIM6 through immunohistochemical staining. The correlation between TRIM6 and patients’ characteristics was assessed by Chi-square test. Log-rank test and Cox hazard regression test were conducted for univariate and multivariate survival analyses, respectively. Two human HCC cell lines, Huh7 and Hep3B, were subjected for knockdown and overexpression assays, followed by phonotype tests including proliferation and invasion. Nude mice were used to generate xenograft model to validate our findings in vivo.

**Results:**

TRIM6 was highly expressed in HCC specimen compared to nontumorous liver tissues. Higher TRIM6 expression was correlated with larger tumor size, later tumor stage, and worse prognosis. According to the cellular experiments, TRIM6-knockdown resulted in decreased expression of cyclin B1, c-Myc, Snail, MMP2, and VEGF-A. Consistently, TRIM6-knockdown led to impaired HCC proliferation, invasion, and angiogenesis. In contrast, TRIM6 overexpression showed opposite effects. Finally, the oncogenic role of TRIM6 in HCC was validated by in vivo mice experiments.

**Conclusion:**

TRIM6 can serve as a novel prognostic factor for HCC, which functions by multiple signaling pathways.

## 1. Introduction

Hepatocellular carcinoma (HCC) ranks among the mostprevalent malignancies worldwide and the third leadingcause of cancer-related death [1]. Despite remarkableprogress in the treatment, such as radiochemotherapy andtargeted therapies, surgical resection remains the mostrecommended strategy. Nevertheless, a large number ofHCC patients suffer with disease recurrence and metastasis [2]. Currently, the 5-year overall survival rate of HCC isapproximately 20% [3]. Thus, it is imperative to identifymore effective treatment strategies through a deeperunderstanding of the molecular mechanisms underlyingHCC progression.

The TRIM (tripartite motif-containing) protein family is characterized by an amino-terminal RBBC (RING-B-box-coiled-coil) domain structure, which is highly conserved in different species. Till now, more than 80 human TRIM proteins have been identified. TRIM proteins exert distinct biological functions in numerous tissues and organs. For example, several TRIM proteins (TRIM5, TRIM15, and TRIM22) were reported to play roles in innate immune response and possess anti-HIV activity [4]. Meanwhile, some TRIM proteins act as platforms during assembling of autophagy regulators, including TRIM13, TRIM21, TRIM28, etc. [5–7].

Interestingly, TRIM proteins were reported to play complicated roles in malignancies. On one hand, several TRIMs promote oncogenic process. For example, TRIM25 has been implicated to enhance proliferation of breast cancer and ovarian cancer [8, 9]. TRIM44 overexpression also results in malignant outcomes of gastric cancer and breast cancer [10, 11]. On the other hand, certain TRIM proteins were reported to serve tumor-suppressive functions. For instance, TRIM3 and TRIM26 were lower expressed in HCC, and their reduced expression was correlated with aggressive tumor growth [12, 13]. Similarly, TRIM62 was reported as a tumor suppressor in breast cancer and lung cancer, which can regulates cell polarity and epithelial plasticity [14, 15]. Even the same TRIM protein may play diverse roles in different tumor types. The TRIM16, for instance, induces HCC migration and invasion [11], while inhibiting proliferation of neuroblastoma and breast cancer [16, 17].

Here in the current study, we mapped the protein expression pattern of TRIM6 in HCC and investigated its clinical significance on predicting patients’ prognoses. Meanwhile, we conducted in vitro and in vivo experiments to confirm the oncogenic role of TRIM6 in HCC progression. Finally, the underlying molecular mechanism of TRIM6 in HCC was elucidated.

## 2. Materials and methods

### 2.1. Patients and samples

This study enrolled a retrospective HCC cohort that underwent surgical resection in The Second Affiliated Hospital of Shandong First Medical University. All HCC tissues were formalin-fixed paraffin-embedded (FFPE) and were used for immunohistochemistry analyses. No patient received any adjuvant therapy before surgery. All patients in this cohort were pathologically diagnosed with HCC and have intact follow-up information.

### 2.2. Immunohistochemistry (IHC) staining

IHC experiments were conducted according to the standard procedures. Briefly, FFPE HCC tissue samples were cut into 4–6-μm sections, which were dried and deparaffinized. After antigen retrieval, sections were blocked to prevent unspecific staining reaction, followed by incubating with primary TRIM6 antibody at 4 °C overnight. The next day, slides were washed and incubated with secondary antibody. Then 3,3-diaminobenzidine (DAB) reaction buffer was used for staining visualization. The staining score was evaluated based on both the staining intensity (scored 0, 1, 2, 3) and percentage of positively stained cells (scored 0–100). According to the median IHC score, all patients were grouped into the low-TRIM6 group (n = 69) or the high-TRIM6 group (n = 69).

### 2.3. Cell culture and stable cell line

Two human HCC cell lines, Huh7 and Hep3B, were obtained from the Cell Bank of the Institute of Biochemistry and Cell Biology (China). Both cell lines were cultured at 37 °C with a humidified atmosphere containing 5% CO_2_, using DMEM containing 10% fetal bovine serum (FBS) and 1% penicillin/streptomycin.

Short hairpin RNA (shRNA) targeting TRIM6 and scrambled control sequence were synthesized and inserted into the pLKO.1-puro vector as described by others [18]. The human TRIM6 cDNA was subcloned into the pEZ-Lv201 vector. Lentivirus production and cell infection were conducted according to the manufactures’ instructions to establish stable cell lines.

### 2.4. Western blot

Cultured cells were lysed using RIPA (Beyotime, China) buffer containing phosphatase inhibitor and protease inhibitor cocktail (Roche, USA). After denatured, a total amount of 20 μg proteins were subjected to SDS-PAGE electrophoresis, and then transferred to polyvinylidene difluoride (PVDF) membranes (Millipore, USA). Then the membranes were blocked and incubated with specific antibodies (1:1000 dilution) at 4 °C overnight, followed by incubation with HRP-conjugated secondary antibodies (1:10,000 dilution) for another 1 h at room temperature. Finally, immunoblotting results were visualized with the chemiluminescent (ECL) substrate. The primary antibodies were anti-TRIM6 (#SAB1306751, Sigma-Aldrich), anti-Snail (#3879, Cell Signaling Technology), anti-MMP2 (#40994, Cell Signaling Technology), anti-Cyclin B1 (#12231, Cell Signaling Technology), anti-c-Myc (#18583, Cell Signaling Technology), anti-beta-actin (#4970, Cell Signaling Technology). The secondary antibodies were antirabbit IgG (#7074, Cell Signaling Technology) and antimouse IgG (#7076, Cell Signaling Technology).

### 2.5. Proliferation assay

Cell Counting Kit-8 (CCK-8) assay method was used to detect cell viability and proliferation. The stable cell lines (100 μL) were seeded into the 96-well plate at a density of 4000 cells/well in triplicate and cultured in 37 °C incubator. At each designated time point (12 h, 24 h, 36 h, 48 h, 60 h), 10 μL CCK-8 reagent was added into each well, followed by incubation for another 1 h in an incubator at 37 °C. Afterwards, the OD values were detected at 450 nm using a microplate reader. Each experiment was repeated 3 times.

### 2.6. Invasion assay

Matrigel–Transwell assay was conducted to test the invasion capacity of transfected cells. Cells were seeded in the upper chamber of the transwell apparatus (8 μm) precoated with Matrigel (Cat. #354230, BD Biosciences, USA), and were cultured in serum-free DMEM medium after cell adhesion. The lower side of the chamber was enhanced with complete DMEM containing 10% FBS as a chemoattractant. After 48 h of culturing, noninvaded cells on the upper chamber were gently removed, while the remaining cells were fixed and stained with crystal violet. Three randomly selected visual fields were pictured under a light microscope to count the invaded cells.

### 2.7. ELISA

Stable cells were seeded in 6-well plates at a density of 80,000 cells/well and cultured in complete DMEM medium containing 10% FBS for 8 h to allow cell adhesion. Then the medium were replaced with serum-free medium and cultured in 37 °C incubator. After 48-h incubation, the medium was collected and centrifuged to collect supernatant. VEGF-A protein in the medium supernatant was further tested using the ELISA kit (#BMS277-2, Thermo Fisher Scientific) according to the protocol of the manufacture. The VEGF-A level was averaged based on the cell number in each group.

### 2.8. Mouse model

BALB/c 4-week-old nude mice were purchased from the Shanghai Laboratory Animal Center. The xenograft model was established by subcutaneously injecting stable Hep3B cells into the nude mice. Tumor diameter was measured by a Vernier caliper every 5 days, and the tumor volume was calculated using the formula: (length×width×width)/2. After 1 month, all mice were scarified to isolate the xenografts.

### 2.9. Statistics

IBM SPSS Statistics 22.0 software, GraphPad Prism 9.0 software, and ImageJ software were used for statistical analyses. Significance was evaluated using two-tailed Student’s t-test for two-group comparisons or one-way ANOVA for multiple comparisons. Clinical data were analyzed using the chi-square test, Kaplan–Meier test, and Cox hazard regression test. p < 0.05 was considered statistically significant.

### 2.10. Ethics

The Research Ethics Committee of the Second Affiliated Hospital of Shandong First Medical University reviewed and approved all protocols involving human specimens and animal experiments (No. 2022-095). Written informed consent was obtained from each participant or a direct relative.

## 3. Results

### 3.1. Patient information

Clinicopathological characteristics of enrolled cases were listed in Table 1. The median age of our retrospective cohort is 67 years old at the time of diagnosis, ranging 39–84 years old. There were only 35 females, whereas there were 103 males. Up to 104 cases were confirmed as tumor location in right liver lobe, 30 cases at left liver lobe, while the other 4 cases with bilateral tumor location. The median tumor size was 4.9 cm, ranging 0.6–17.0 cm in diameter. As for the differentiation grade, 41 cases were diagnosed with well differentiation (grade I), 62 cases with moderate differentiation (grade II), 31 cases with poor differentiation (grade III), and the other 4 cases were undifferentiated (grade IV). According to the AJCC 7^th^ edition staging system, 80 cases were classified as TNM stage I, 26 cases as stage II, and 32 cases as stage III.

### 3.2. High expression of TRIM6 in HCC

**Figure 1:**
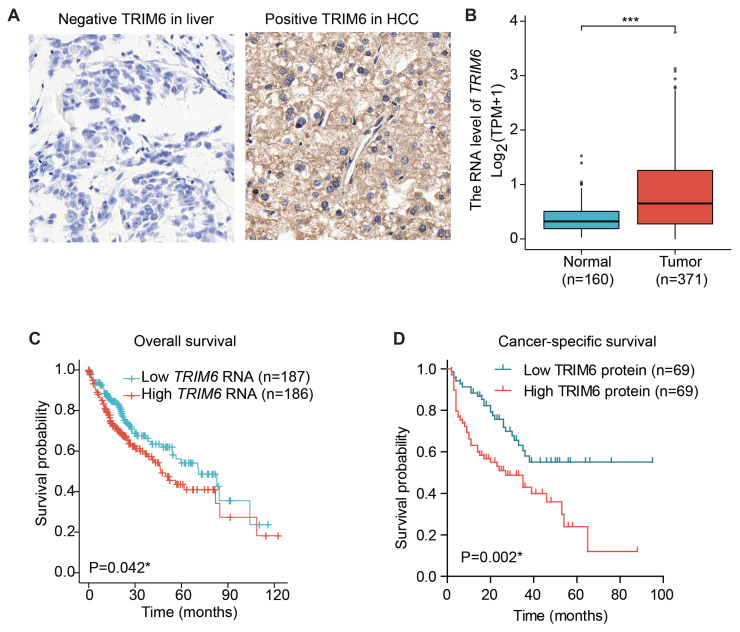
Expression and prognostic role of TRIM6 in HCC. (A) Immunohistochemical staining showed representative negative TRIM6 in normal liver tissues and high TRIM6 in HCC specimen. Magnification:400X. (B) The mRNA level of *TRIM6* in HCC and normal liver tissues were extracted from TCGA dataset, which showed an elevated *TRIM6* in HCC. (C) TCGA dataset showed that patients with higher *TRIM6* mRNA level exhibited worse overall survival. (D) Kaplan–Meier analysis of our retrospective cohort indicated that higher protein expression of TRIM6 in HCC specimen indicated worse cancer-specific survival.

By IHC analyses, we found that TRIM6 protein was highly expressed in HCC specimen, while the staining intensity was weaker in nontumorous liver tissues (Figure 1a). Consistently, TCGA dataset showed a significantly higher mRNA level of TRIM6 in HCC tumor tissues than that in normal liver tissues (Figure 1b, p < 0.001). Therefore, we followed this up by analyzing whether TRIM6 expression has any correlation with patients’ characteristics (Table 1). Chi-square test revealed that tumors with larger tumor size exhibited higher TRIM6 level (p = 0.041). Additionally, TRIM6 protein level was positively correlated with TNM stage (p = 0.005). In contrast, statistical analysis did not find any significant correlation between TRIM6 and other variables (p > 0.05).

### 3.3. High TRIM6 can predict worse HCC prognosis

**Figure 2:**
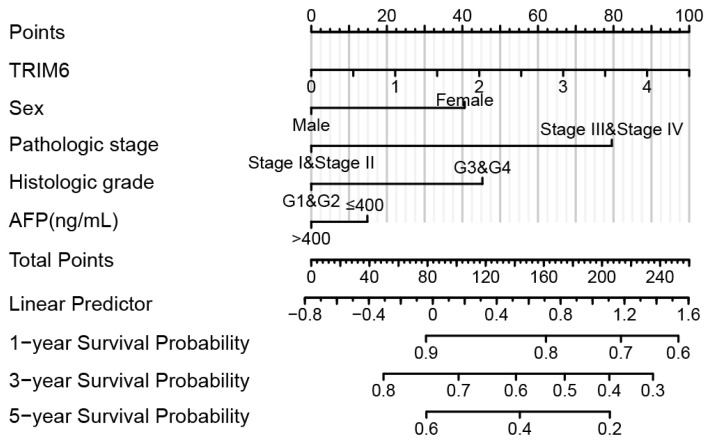
HCC prognostic predictive nomogram generated using the data from TCGA datasets. Overall survival nomogram was established based on the patients’ information from TCGA datasets.

Interestingly, TCGA dataset not only showed a higher TRIM6 level in HCC, but also implied the prognostic significance of TRIM6. In detail, patients with lower TRIM6 mRNA levels had a better overall survival rate compared to those with higher TRIM6 mRNA levels (Figure 1C, p = 0.042). To further investigate its clinical relevance, we also conducted a survival analysis of our retrospective cohort based on the protein level of TRIM6 (Figure 1d). It was noted that higher TRIM6 protein expression in tumor tissues is correlated with worse cancer-specific survival of HCC patients (p = 0.002). The 3-year cancer-specific survival rate of patients with low-TRIM6 expression was 58.0%, while it decreased to 43.0% in those with high-TRIM6 expression. Furthermore, TRIM6 exhibited a better prognostic predictive role in combination with other conventional prognostic factors such as tumor stage, differentiation grade, serum AFP level, etc. (Figure 2).

**Figure 3:**
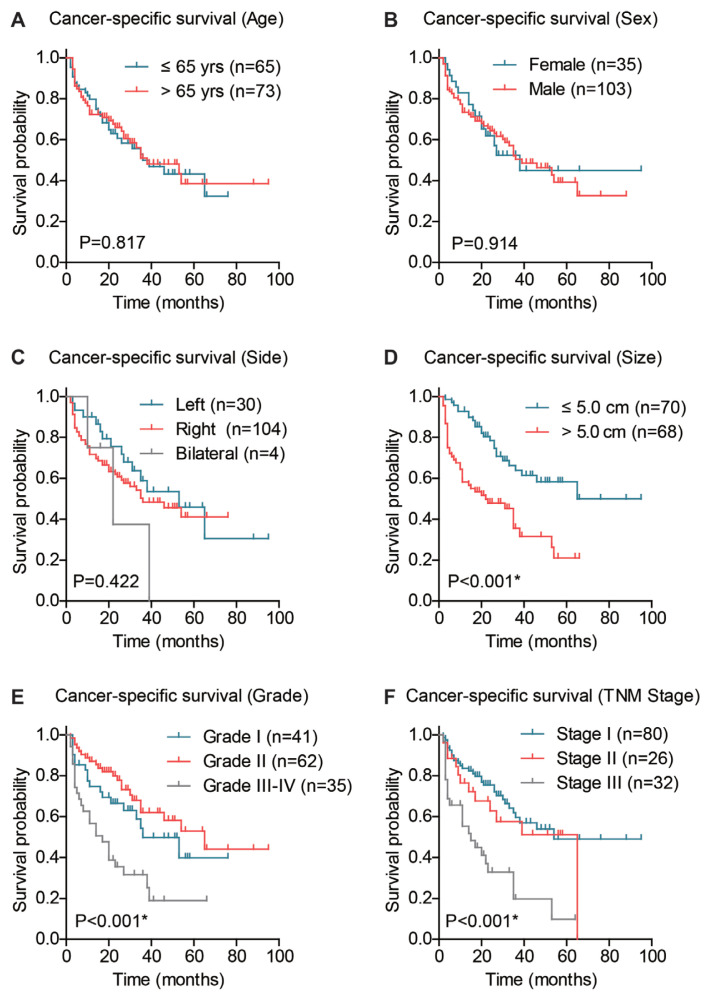
Kaplan–Meier survival analyses based on patients’ clinicopathological characteristics. The cancer-specific survival of HCC patients were analyzed by Kaplan–Meier method and log-rank test based on patients’ age (A), sex (B), tumor location (C), tumor size (D), tumor differentiation grade (E), and TNM stage (F).

The significance of other prognostic factors were also evaluated using the Kaplan–Meier method (Table 2). Accordingly, patients’ age, sex, and tumor location did not show any statistically significant effect on the cancer-specific survival of HCC (Figures 3a–3c, p > 0.05). As expected, larger tumor size was significantly correlated with worse prognosis (median survival time 22.0 months versus 65.0 months, Figure 3d, p < 0.001). Meanwhile, patients with poor differentiated or undifferentiated grade exhibited a lower 3-year survival rate (31.6%) than those with better differentiation grade (49.8% and 62.1%, Figure 3e, p < 0.001). Another conventional prognostic factor is TNM stage. The 3-year survival rates of patients with TNM stage I, II, III were 59.5%, 57.6%, and 19.8%, respectively (Figure 3f, p < 0.001).

Cox hazard regression analysis was next conducted using all the significant variables above to evaluate their independent prognostic effect (Table 3). Tumor size and TNM stage were both confirmed as independent prognostic factors (p = 0.034 and p = 0.049, respectively). Of note, TRIM6 was also validated as a novel independent prognostic biomarker for HCC prognosis (hazard ratio 1.788, 95% confidence interval 1.062–3.010, p = 0.029).

### 3.4. TRIM6 promotes HCC progression through multiple signaling pathways

**Figure 4:**
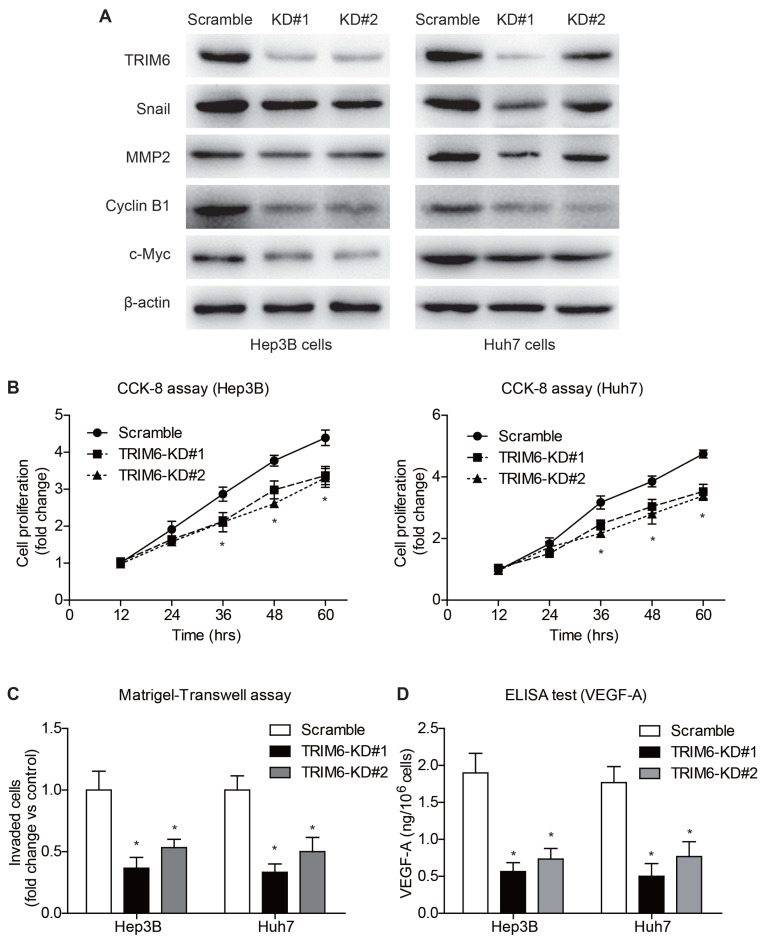
TRIM6 knockdown attenuated HCC proliferation, invasion, and angiogenesis. (A) Immunoblotting data validated the knockdown (KD) efficiency of shRNAs targeting TRIM6, using scrambled shRNA as control. In both cell lines, the expression of Snail, MMP2, cyclin B1, and c-Myc were decreased after TRIM6-knockdown (B) CCK-8 analyses revealed that TRIM6-knockdown impaired the proliferation viability of both Hep3B and Huh7 cells. (C) As indicated by the Matrigel–Transwell assay, knocking-down TRIM6 inhibited the invasion capacity of HCC cells. (D) The secreted level of VEGF-A in the cell culturing medium was decreased in TRIM6-knockdown cells.

Considering the clinical significance of TRIM6, we were engaged to further explore its detailed tumor-related functions in HCC. We firstly established stable TRIM6-knockdown cells using Hep3B and Huh7 cell lines, respectively (Figure 4a). After validating the knockdown efficiency, cell lines were subjected to CCK-8 assays to test their proliferation viability (Figure 4b). In both cell lines, TRIM6-knockdown cells exhibited impaired proliferation capacity compared to those infected with scrambled-shRNA. Besides proliferation, TRIM6-knockdown resulted in decreased invasion capacity of HCC cells as revealed by Matrigel–Transwell experiments (Figure 4c). Consistent with the phenotypes, immunoblotting revealed that TRIM6-knockdown can inhibit the expression of c-Myc, Cyclin B1, MMP2, and Snail. In addition, ELISA data showed that silencing TRIM6 resulted in decreased basal secretion of VEGF-A in the cell culturing medium supernatant (Figure 4d).

**Figure 5:**
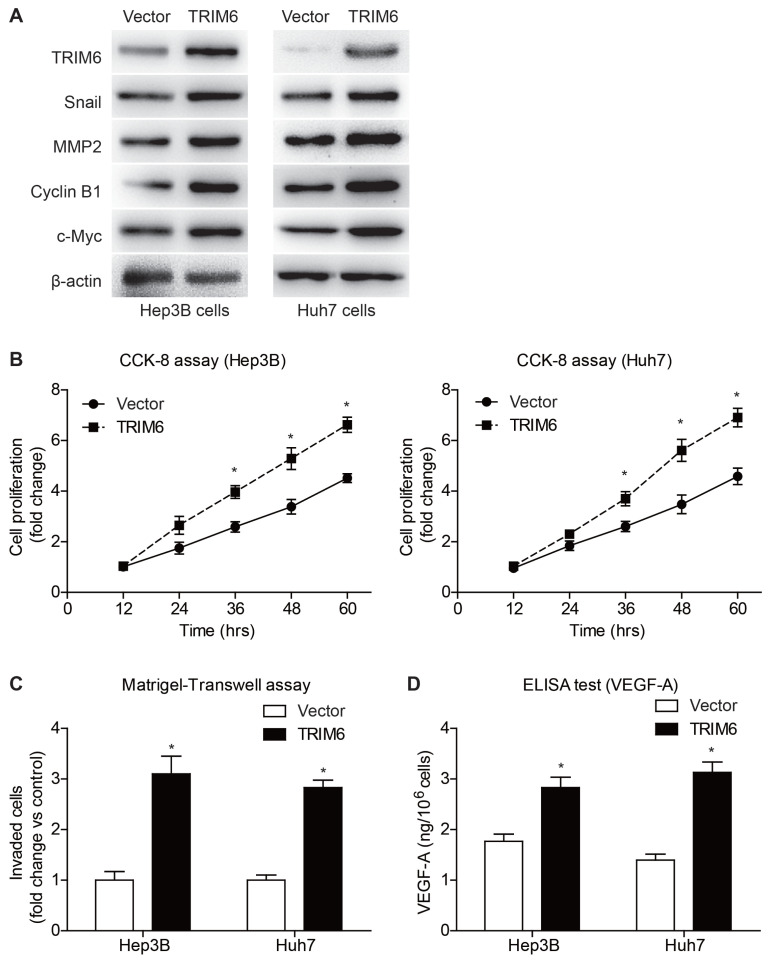
Overexpressing TRIM6 enhanced HCC proliferation, invasion, and angiogenesis. (A) The protein levels of TRIM6, Snail, MMP2, cyclin B1, and c-Myc were tested in stable-infected cell lines. (B) CCK-8 data showed that overexpressing TRIM6 can promote the proliferation of HCC cells. (C) Matrigel–Transwell experiments revealed enhanced tumor invasion capacity after overexpressing TRIM6 in HCC. (D) The secretion of VEGF-A in cell culturing medium was increased in TRIM6-overexpression group compared to control cells.

On the other hand, overexpressing TRIM6 resulted in up-regulated expression of c-Myc, Cyclin B1, MMP2, and Snail (Figure 5a). Moreover, TRIM6 overexpression led to enhanced cell proliferation and invasion capacities (Figures 5b and 5c). In contrast with the effect of TRIM6-knockdown, its overexpression resulted in higher VEGF-A secretion in the HCC cell culture medium (Figure 5d).

**Figure 6:**
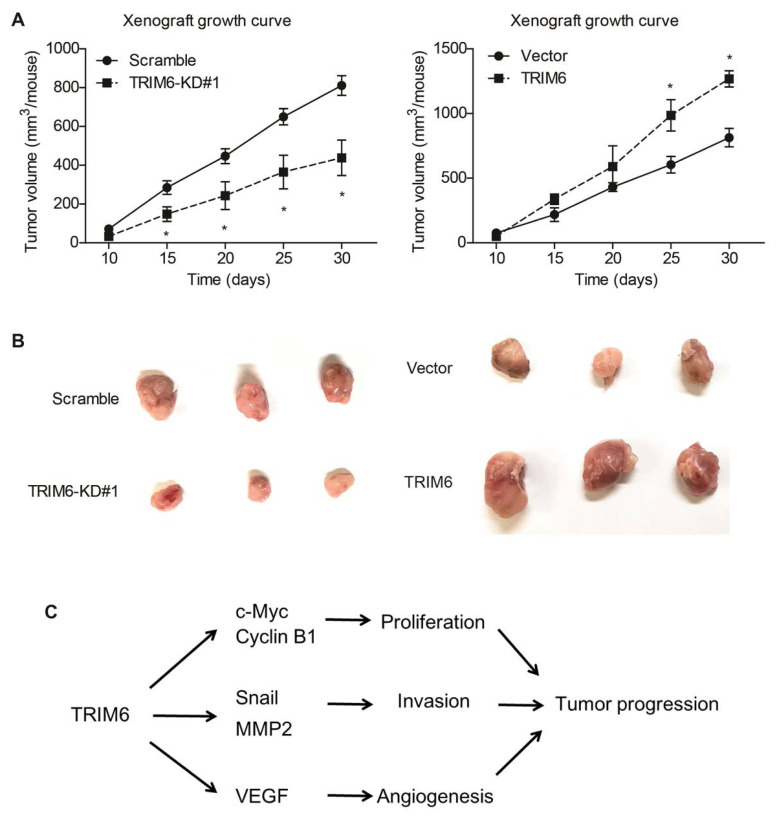
TRIM6 promoted HCC growth in vivo. (A) Growth curve of xenografts were plotted in mice model, including both the TRIM6-knockdown (KD) group and TRIM6-overexpression group. (B) Isolated xenografts were pictured after mice sacrifice. (C) Schematic explanation on how TRIM6 promotes HCC progression from multiple signaling pathways.

Finally, we conducted in vivo analysis using nude mice to establish xenograft model. By monitoring the xenograft growth curve, we confirmed that silencing TRIM6 attenuated HCC growth (Figures 6a and 6b). Therefore, we came to our major conclusion that TRIM6 can promote HCC progression through multiple signaling pathways (Figure 6c).

## 4. Discussion

Despite TRIM6 having been reported to be involved malignancies, its detailed function seems distinct in different tumor types. We initially found that TRIM6 was more highly expressed in HCC tissues compared with normal liver tissues from both mRNA and protein levels. A similar finding was previously reported in colorectal cancer tissues by Zheng et al [18], which exhibited an upregulated TRIM6 expression than adjacent colon tissues. A recent bioinformatics study systematically analyzed the expression profiles of TRIM protein family members in HCC [19]. Based on the Oncomine and UALCAN datasets, TRIM6 was significantly elevated in HCC versus normal tissues on its mRNA level. Consistently, another study also revealed remarkably higher TRIM6-mRNA level in HCC than normal tissues according to Wurmbach’s dataset [20].

The dysregulated expression prompted us to investigate its correlations with clinicopathological characteristics and prognoses of HCC patients. Based on statistical analyses, we found that expression level of TRIM6 was positively correlated with HCC tumor size and TNM stage. Furthermore, patients with higher TRIM6 level were characterized both with worse overall survival and worse cancer-specific survival. Consistent with our findings, bioinformatics data revealed that glioma patients with higher TRIM6 level experience worse prognoses [21, 22]. Of note, both our data and others’ paper confirmed that combination of TRIM6 with other prognostic factors can improve its prognostic predictive capacity, highlighting its potential in clinical practice.

As for the molecular mechanisms, Wei and his colleagues reported that TRIM6 functions by positively regulating the signaling of YAP1 (Yes-associated Protein 1), a well-known oncogenic driver, in a STUB1 (stress induced phosphoprotein 1 homology and U-box containing protein 1)-dependent manner [23]. Also, Zheng et al. revealed that TRIM6-knockdown induced cell cycle arrest at G2/M phase of colon cancer cells by decreasing the ubiquitination level of TIS21 [18]. In our study, immunoblotting results identified elevated expression of c-Myc and cyclin B1, two critical proteins in cell proliferation, after overexpressing TRIM6 in HCC cell lines. Indeed, overexpressing TRIM6 resulted in accelerated HCC cell proliferation, while silencing TRIM6 led to an attenuated proliferation speed. In addition, we conducted in vivo assays using xenograft implantation in nude mice model, thus providing the first in vivo evidence of TRIM6’s role in promoting HCC growth. Briefly, overexpressing TRIM6 led to accelerated xenograft growth while silencing TRIM6 inhibited xenograft growth.

Another downstream effector of TRIM6 in colorectal cancer is STAT3 (signal transducer and activator of transcription 3). As reported by Zhao et al., TRIM6 can upregulate the activity of STAT3 via the suppressor of cytokine signaling 2 (SOCS2) [24]. Through this signaling pathway, TRIM6 can enhance the migration and metastasis of colorectal cancer. Interestingly, our data also identified that overexpressing TRIM6 promotes HCC invasion, while silencing TRIM6 inhibits HCC invasion. However, we did not find any statistically significant alteration of STAT3 or phosphor-STAT3 in HCC cell lines (data not shown). Instead, the protein expression levels of Snail and MMP2 were positively regulated by TRIM6, which may subsequently modulate the HCC invasion. Notably, we recognized for the first time that the autocrine VEGF-A in cell culture medium was elevated upon TRIM6-overexpression, implying TRIM6 may also participate in the angiogenesis process during HCC progression. Besides tumor correlation, TRIM6 also participates in innate immunity regulation and is reported to be a driver in dermatomyositis. From this aspect, identifying the dysregulated expression of TRIM6 may be more valuable for tumor patients combined with immune diseases [25, 26]. Furthermore, Su and his colleagues assessed the correlation between TRIM6 and immune cell infiltration in HCC [19]. As a result, TRIM6 has a significant correlation with B cell infiltration, implying that TRIM6 may participate in tumor immunology response. However, the detailed immunology role and functional mechanisms of TRIM6 in HCC progression require further investigation.

There are several limitations of our study. Firstly, clinical data were obtained from a single medical center, which may contain regional bias. We tried to make our major conclusion more convincible by retrieving the data from TCGA datasets as well as previous studies. Secondly, knockdown of TRIM6 has been reported to increase the sensitivity to 5-fluorouracil and oxaliplatin in colorectal cancer [18]. Here we did not explore the correlation between TRIM6 with chemo-resistance due to incomplete patients’ information. We will further dig into the detailed crosstalk between TRIM6 and drug resistance, which is critical for targeted therapy development. Finally, the upstream regulators of TRIM6 in HCC need to be elucidated. A recent study reported a NEAT1-miR-101-3p/335-5p/374a-3p/628-5p-TRIM6 network in lung adenocarcinoma, whose hyper-activation results in upregulated TRIM6 level and poorer prognosis [27]. Nevertheless, their data was predominantly based on in silico analyses, which need further experimental validation.

## 5. Conclusion

Higher TRIM6 expression promotes HCC progression and indicates unfavorable prognosis. Our data not only identifies TRIM6 as a novel prognostic biomarker, but also highlights its significance as a potential therapeutic target for HCC.

## Figures and Tables

**Table 1:** Characteristics of HCC patients and their relationships with TRIM6 level.

Variables	Cases	TRIM6 protein expression		p-value
	(n = 138)	Low (n = 69)	High (n = 69)	
**Age**				0.394
<65 years	65	30	35 (53.8%)	
>65 years	73	39	34 (46.6%)	
**Sex**				0.328
Female	35	20	15 (42.9%)	
Male	103	49	54 (52.4%)	
**Tumor side**				0.525
Left lobe	30	14	16 (53.3%)	
Right lobe	104	54	50 (48.1%)	
Bilateral	4	1	3 (75.0%)	
**Tumor size**				0.041*
≤5.0 cm	70	41	29 (41.4%)	
>5.0 cm	68	28	40 (58.8%)	
**Differentiation**				0.373
Grade I	41	24	17 (41.5%)	
Grade II	62	30	32 (51.6%)	
Grade III–IV	35	15	20 (57.1%)	
**TNM stage**				0.005*
Stage I	80	49	31 (38.8%)	
Stage II	26	11	15 (57.7%)	
Stage III	32	9	23 (71.9%)	

Note:

* p < 0.05 by chi-square test.

Abbreviations: HCC, hepatocellular carcinoma; TRIM6, Tripartite Motif Containing 6.

**Table 2:** Prognosis of HCC patients by Kaplan–Meier analyses.

Variables	Cases	CSS (months)		p-value
	(n=138)	Median	3-year DFS	
**Age**				0.817
≤65 years	65	36.0	49.9%	
>65 years	73	38.0	51.0%	
**Sex**				0.914
Female	35	38.0	52.4%	
Male	103	39.0	50.4%	
**Tumor side**				0.422
Left lobe	30	53.0	58.8%	
Right lobe	104	36.0	48.3%	
Bilateral	4	22.0	37.5%	
**Tumor size**				
<5.0 cm	70	65.0	63.9%	<0.001*
>5.0 cm	68	22.0	35.6%	
**Differentiation**				<0.001*
Grade I	41	36.0	49.8%	
Grade II	62	65.0	62.1%	
Grade III–IV	35	17.0	31.6%	
**TNM stage**				<0.001*
Stage I	80	54.0	59.5%	
Stage II	26	65.0	57.6%	
Stage III	32	15.0	19.8%	
**TRIM6 expression**				0.002*
Low	69	Unavailable	58.0%	
High	69	27.0	43.0%	

Note:

* p < 0.05 by log rank test.

Abbreviations: HCC, hepatocellular carcinoma; CSS, cancer-specific survival.

**Table 3:** Cox multivariate analysis of HCC prognosis.

Variables	Hazard ratio	95% confidence interval	p-value
**Tumor size**	1.876	1.050–3.352	0.034*
**Differentiation grade**	1.403	0.973–2.022	0.070
**TNM stage**	1.348	1.002–1.814	0.049*
**TRIM6 expression**	1.788	1.062–3.010	0.029*

Note:

* p < 0.05 by Cox hazard regression test.

Abbreviations: HCC, hepatocellular carcinoma; TRIM6, tripartite motif containing 6.
